# Evaluating the Impact of a School-Based Youth-Led Health Education Program for Adolescent Females in Mumbai, India

**DOI:** 10.5334/aogh.2791

**Published:** 2020-08-26

**Authors:** Priya Shankar, Dana Sievers, Ricky Sharma

**Affiliations:** 1 Department of Pediatrics, University of California San Francisco, San Francisco, CA, US; 2 Harvard T.H. Chan School of Public Health, Boston, MA, US; 3 Girls Health Champions, San Francisco, CA, US

## Abstract

**Background:**

India’s 120 million adolescent girls often have limited opportunities to receive health education, as health-related content in school curricula can be minimal, and the few existing external interventions for this demographic rarely cover multiple topics.

**Objectives:**

This study conducted a program evaluation of Girls Health Champions, a school-based peer education intervention in Mumbai, India that educates girls about leading causes of adolescent morbidity and mortality, including nutrition, mental health, and sexual & reproductive health.

**Methods:**

Female participants ages 12 to 16 in the eighth, ninth, and tenth standards were recruited at five participating schools in Mumbai, India to learn a multi-topic health curriculum from their peers, with a subset of ninth standard participants in each school trained as the peer educators. Using a quasi-experimental design, participant survey data was collected three times during the 2016–2017 academic year: at baseline, immediately following the peer-led education sessions, and five months following these sessions. Outcomes of interest included change in knowledge levels and health attitudes following the intervention, as well as retention at mid-year. An additional outcome was the change in self-reported leadership skills of peer educators before and after participating.

**Findings:**

Compared to baseline, participants demonstrated statistically significant increases in knowledge levels (+48%, p < 0.001) and positive shifts in health-related attitudes (+42%, p < 0.001). These changes were maintained at mid-year (+29% for knowledge levels, p < 0.001; +37% for attitudes, p < 0.001). Findings were consistent when data was stratified by standard and peer educator status (peer educators versus non-peer educators). Peer educators also demonstrated a statistically significant increase in their interest in health promotion.

**Conclusions:**

This study demonstrates the effectiveness of the peer education delivery model and finds school-based, peer-led programs covering a range of adolescent health topics can significantly increase knowledge and shift attitudes of program participants. Such benefits can accrue to both peer educators and non-peer educator program participants.

## Introduction

India has the world’s largest adolescent population, which includes 120 million adolescent girls [1,2]. Many females encounter a variety of gender-related health challenges during adolescence and early adulthood, including inadequate nutrition, iron-deficiency anemia, mental illness, gender-based violence, menstruation-related difficulties, and teen pregnancy [3].

More than half of Indian adolescent girls are estimated to suffer from anemia [4]. Many girls in the adolescent age range eat well below the minimum recommended daily caloric intake [5]. Additionally, many girls forego taking free and widely available iron and folic acid tablet supplementation as a result of a lack of knowledge about their importance or undue concern regarding side effects [6].

Mental health ailments are the leading cause of disease burden among adolescents in India, with self-harm or suicide being the leading causes of death for girls ages 15 to 19 [7]. Academic and social pressures at schools, along with limited awareness regarding stress, anxiety, and depression, result in girls lacking tools to identify or mitigate stressors [8].

Nearly a third of adolescent girls in India will experience sexual abuse or become victims of gender-based violence in their lifetimes [9]. Additionally, studies show females internalize notions related to violence such that 52% of women believe it is acceptable for men, under certain circumstances, to hit their wives [10].

Many girls have limited knowledge about menstruation at the time of their first period, with infections during menstruation common due to a lack of menstrual hygiene and preparedness [11,12]. Only 58% of females ages 15 to 24 nationwide report using hygienic methods of menstrual protection [10].

One-fifth of Indian females are estimated to experience childbearing during adolescence [13,14]. Additionally, young adults ages 15 to 29 are responsible for 50% of the new cases of HIV occurring each year in India [15].

Mumbai is one of India’s largest cities, with more than 40% of its residents living in slums [16]. District-level data suggests that in urban portions of Mumbai, nearly half of women ages 15 to 49 years of age are anemic and approximately 20% are underweight. One in ten women are married before 18 years of age, with only 27.7% of women reporting having had a female health worker speak to them about family planning [10]. Additionally, mental health issues are a challenge in slum communities, with studies suggesting such communities face a disproportionately higher burden of common mental disorders [17].

The Indian government has identified peer health education as a potential means for addressing these health challenges of adolescence and early adulthood [18]. Peer education is regarded to be a cost-effective, context-sensitive way to deliver health training [19]. The peer-to-peer approach is considered an effective health promotion strategy for the following reasons: 1) timing: adolescents are at a critical juncture in life, such that the ideas, notions, and habits formed during this period can influence the course of their adulthood; and 2) receptivity: peer educators can be viewed as credible, reliable and socially accepted role models for engaging youth [20]. Though peer education models have been implemented throughout the world, few of them have been rigorously assessed or implemented in schools.

## Methods

### Study Design

This study was a program evaluation, using a quasi-experimental design, that sought to assess the effectiveness of Girls Health Champions, a school-based peer health education intervention in Mumbai, India for adolescent girls in the eighth, ninth, and tenth standards (grades). Research was conducted between August 2016 and January 2017 in five government-aided schools in low-income urban communities in Mumbai. The intervention was conducted separately at each school during this period, utilizing the same protocol in each school.

The intervention involved first educating and training a subset of adolescent girls at each participating school as peer health educators (Champions) using the four-part Girls Health Champions curriculum. Following the completion of this training, the Champions were then provided structured opportunities in-school to teach the same four-part curriculum directly to their classmates in the eighth, ninth, and tenth standards. For data collection, the researchers administered three evaluation tools (Knowledge, Attitudes, and Leadership Skills Assessments) before and after the intervention. The researchers also re-administered the Knowledge and Attitudes Assessments five months following the intervention to evaluate retention.

### Intervention Overview

The Girls Health Champions program features four modules covering many of the aforementioned leading causes of adolescent morbidity and mortality in India, including: nutrition & anemia, mental health & gender-based violence, menstruation, and sexual & reproductive health. Modules were developed in collaboration with pediatricians, obstetricians/gynecologists, psychiatrists, psychologists, and educational experts from India and the United States. The modules include educational content and corresponding interactive activities to solidify and contextualize foundational knowledge.

In the first module, Nutrition & Anemia, youth learn about signs and symptoms of iron-deficiency anemia as well as ways to diagnose and treat it through iron and folic acid tablet supplementation. Additionally, youth practice calculating Body Mass Index and learn the components of a healthy, balanced diet. This module also contextualizes the science of nutrition and anemia with corresponding population-level statistics about anemia and prevalence of undernutrition in India and its overwhelming impact on young girls and women.

In the second module, Healthy Minds and Safe Bodies, girls learn about self-esteem, body image, resilience, and common mental illnesses such as anxiety and depression. Much of this module focuses on shifting common negative attitudes towards individuals with mental illness and understanding potential treatment options. The module further seeks to reduce stigma and incorporates therapeutic exercises. Additionally, girls learn about gender-based violence and are provided with techniques to protect themselves against violence and maintain personal safety.

In the third module, Menstrual Health, girls learn about female reproductive anatomy, the biology surrounding menstruation, and ways to ensure a safe and hygienic menstrual cycle. Girls also learn about common stigmas and taboos surrounding menstruation and are asked to form their own opinions regarding these practices in their communities.

In the final module, Reproductive Health, girls learn about the anatomy and biology of reproduction, as well as ways to prevent pregnancy through modes of protection ranging from abstinence to oral contraceptive utilization. Girls learn about the adverse consequences of early marriage, rates of child marriage in their communities, and the ways in which youth are attempting to shift attitudes about these topics. Girls are also given comprehensive information about the leading causes of Sexually Transmitted Infections (STIs) such as HIV, gonorrhea, chlamydia, and syphilis.

### Participant Selection

The selection of the five participating schools was done on a convenience basis, with sites being chosen in partnership with Niramaya Health Foundation (NHF), a Mumbai-based community health organization. Schools were selected in several low-income communities in Mumbai. Prior to beginning the intervention in participating schools, the researchers reviewed each school’s science curriculum and noted that such curricula contained minimal to no overlapping content to the Girls Health Champions curriculum.

All female students in the eighth, ninth, and tenth standards who attended participating schools were eligible to participate, with eligible participants ranging between the ages of 12 to 16. From this pool of eligible students, only those students from whom the researchers had obtained both parental consent and child assent and who were present at school on the days of the intervention ultimately participated.

Study participants fell into two main categories: Champions (peer educators) and non-Champions. A cohort of participants were trained as peer health educators, or Champions, at each school. In order to select the Champions, all participating students in the ninth standard at each school were first asked on the child assent form whether they were interested in becoming a Champion peer health educator, which would involve learning and teaching a portion of the Girls Health Champions’ curriculum to their classmates. The intervention was structured to only train a subset of participants (~20 students) at each school as Champions. In schools where more than 20 ninth standard students expressed an interest in becoming Champions, the researchers used random number assignment to select the final roster of Champions from the pool of interested students.

The second main category of participants were the non-Champions. These participants learned the Girls Health Champions curriculum directly from the students at their school who were trained as Champions. Non-Champions participants included the eighth standard and tenth standard students, as well as the ninth standard students who either did not indicate an interest in becoming a Champion or who were not randomly selected from the pool of interested students to become Champions. Demographics between Champions and non-Champions were presumably similar given that all students were from the same school and surrounding communities.

### Intervention Implementation

At each school, following the selection of the Champions, each Champion was provided a printed copy of the entire Girls Health Champions curriculum. The Champions were then divided into four equal-sized groups, with each group assigned to one of the four modules of the Girls Health Champions curriculum. Each of these four groups then separately spent eight hours learning their assigned topic from an NHF adult health worker and practicing the teaching of the module as a group. Over the course of several days, each of the four groups of Champions were then provided structured opportunities during school hours to teach their specific module to the non-Champion participants in their school, as well as to the other three groups of Champions who had been trained in the other three modules. The teaching sessions led by the Champions took four hours in total, and the non-Champion program participants were also provided with a printed copy of the entire curriculum. Five months following the intervention, the Champions taught an abridged version of the modules as a mid-year “refresher” session. This session was conducted in one day at each school and took approximately four hours.

### Ethics Procedures

The researchers received Institutional Review Board approval at the Harvard T.H. Chan School of Public Health (reference number: IRB15-3958). The researchers also received permission from school administrators at participating schools, as well as both parental consent and child assent for all participants. Participation in the program was voluntary.

### Sampling Methods & Data Collection

The study utilized three evaluation tools (Table 1). The Knowledge and Attitudes Assessments were administered to all participants (Champions and non-Champions) at all five schools, while the Leadership Skills Assessment was administered to only the Champions at the five schools. Knowledge was defined as the proportion of questions answered correctly on the Knowledge Assessment, Attitudes as the proportion of statements correctly identifying favorable health attitudes on the Attitudes Assessment, and Leadership as self-reported data from Champions evaluating their personal leadership skills on a five-point scale.

**Table 1:** Evaluation Tools.


**Knowledge Assessment**	20-question assessment focused on measuring participants’ health knowledge 5 questions on each of the 4 modules (nutrition & anemia, mental health/gender-based violence, menstruation, reproductive health) Multiple-choice format Sample questions: “*What is anemia?*” “*Which of the following is part of a female’s reproductive system?*”
**Attitudes Assessment**	6-question assessment focused on measuring participants’ agreement with favorable health attitudes underlying program curricula Agree/disagree format Sample statements: “Individuals with mental illness are crazy.” “Victims of physical or sexual violence are never at fault or to blame.”
**Leadership Skills Assessment**	4-question assessment focused on Champions’ self-perceived leadership skills Five-point Likert Scale format Sample statement: “*I feel confident speaking in front of my classmates*.”


The researchers conducted the intervention and collected participant data at the five participating schools on two separate occasions.

#### August 2016

Champions and non-Champion participants completed the Knowledge and Attitudes Assessments twice: immediately prior to, and immediately following, the intervention. Champions also completed the Leadership Skills Assessment before and after the intervention. The intervention and associated data collection were completed within two weeks at each school.

#### January 2017

Prior to the mid-year “refresher” session, the researchers re-administered the Knowledge and Attitudes assessments to all participants to assess retention from the August 2016 training.

### Data Analysis

The results across the five participating schools were pooled and analyzed in aggregate. For all analyses, statistical significance was defined at the *p* < 0.05 level. All tests were performed using Stata/IC (Version 15, College Station, Texas, 2018).

#### Knowledge & Attitudes Assessment Data

To account for the left-skewness of the data, non-parametric statistical tests were used to assess changes over time and make comparisons between strata. The researchers stratified results by standard, by school, and by Champion status (Champions vs. non-Champions). Given most Champions were in the ninth standard, the researchers performed a sub-analysis controlling for standard that compared results of ninth standard Champions to results of non-Champion ninth standard participants. Furthermore, the researchers stratified Knowledge results by topic, grouping the twenty questions on the Knowledge Assessment into four index variables representing the four modules, and tested for differences in the mean scores across the four modules. To account for the fact that each assessment was repeated with the same subjects, Friedman’s test (the non-parametric version of repeated measures ANOVA) was performed. Although non-parametric tests were used to determine statistical significance, mean results are reported to more clearly reflect differences over time and across strata.

#### Leadership Skills Assessment Data

An average rating was calculated for each question. The distribution of ratings was left-skewed, so nonparametric tests were used to compare average ratings over time.

## Results

### Knowledge Assessment Results

The post-test results (Table 2) show that immediately following the peer-led education sessions, average knowledge scores for participants were nearly 1.5 times the baseline average, with statistically significant gains across all three standards (*ps* < 0.001). Average scores for both Champions and non-Champions significantly improved (*ps* < 0.001), with Champions demonstrating greater improvement (+65%) than non-Champions (+46%).

**Table 2:** Average Knowledge Assessment Scores.

	Pre-Test August 2016 mean % correct (SD)	Post-Test August 2016 mean % correct (SD)	% change vs. Pre-Test (dir./sig.)	Mid-YearRetention January 2017 mean % correct (SD)	% change vs. Pre-Test (dir./sig.)	% change vs. Post-Test (dir./sig.)

All participants	**56.7 (19.8)** N = 738	**84.0 (15.3)** N = 687	+48%***	**72.9 (18.5)** N = 590	+29%***	–13%***
Standard						
*8^th^ Standard*	47.9 (19.8) *n = 162*	74.6 (19.5) *n = 155*	+56%***	65.2 (21.2) *n = 171*	+35%***	–13%***
*9^th^ Standard*	54.1 (19.9) *n = 283*	83.4 (13.5) *n = 258*	+54%***	76.1 (17.7) *n = 263*	+41%***	–9%***
*10^th^ Standard*	64.1 (17.1) *n = 293*	89.9 (10.9) *n = 274*	+41%***	76.1 (13.7) *n = 156*	+19%***	–16%***
Champion status						
*Champion*	49.4 (21.0) *n = 105*	81.5 (15.3) *n = 95*	+65%***	75.0 (21.3) *n = 89*	+52%***	–8%NS
*Non-Champion*	57.9 (19.4) *n = 633*	84.4 (15.3) *n = 592*	+46%***	72.6 (18.0) *n =* 501	+25%***	–14%***
*9^th^ Std. Champion*	50.0 (21.2) *n = 100*	81.0 (15.4) *n = 90*	+62%***	76.3 (21.3) *n = 84*	+53%***	–6%NS
*9^th^ Std. Non-Champion*	56.4 (18.8) *n = 183*	84.7 (12.1) *n = 168*	+50%***	76.1 (15.8) *n = 179*	+35%***	–10%***
Health Topic						
*Nutrition/Anemia*	56.2 (28.5)	87.6 (17.8)	+56%***	75.4 (22.8)	+34%***	–14%***
*Mental Health/Gender-Based Violence*	57.0 (26.4)	80.9 (23.0)	+42%***	69.4 (25.7)	+22%***	–14%***
*Menstruation*	61.5 (25.2)	85.9 (19.2)	+40%***	76.6 (23.8)	+25%***	–11%***
*Reproductive Health*	52.2 (26.4)	81.6 (21.2)	+56%***	70.3 (23.0)	+35%***	–14%***

*Note*: + denotes positive change, – denotes negative change. *** = *p* < 0.001, ** = *p* < 0.01, * = *p* < 0.05, NS = not significant.

At mid-year, after five months had elapsed since the initial intervention, average scores generally decreased when compared to scores immediately post-intervention (*p* < 0.001). One noteworthy exception is the Champions (both in aggregate and the subgroup of ninth Standard Champions), who did *not* experience a statistically significant decline. However, all scores at mid-year demonstrated statistically significant increases when comparing such scores to the baseline scores from five months prior (*ps* < 0.001).

Knowledge varied between topics. At baseline, participants were least familiar with reproductive health. At the post-test, they were most knowledgeable about nutrition & anemia and menstruation; scores on those topics were significantly higher than those in reproductive health and mental health (*p* < 0.05).

At baseline, average scores for all three standards were significantly different from one another (Table 2), with higher scores being associated with higher standard (*p* < 0.001). Champions scored lower than non-Champions, including when comparing ninth standard Champions versus their ninth standard non-Champion counterparts.

### Attitudes Results

At baseline, 46% of participants’ answer choices correctly identified favorable health attitudes on the Attitudes assessment. Scores were similar across all three standards, with Champions demonstrating similar attitudes to non-Champions (Table 3).

**Table 3:** Average Attitudes Assessment Scores.

	Pre-Test August 2016 mean % in agreement (SD)	Post-Test August 2016 mean % in agreement (SD)	% change vs. Pre-Test (dir./sig.)	Mid-Year Retention January 2017 mean % in agreement (SD)	% change vs. Pre-Test (dir./sig.)	% change vs. Post-Test (dir./sig.)

All participants	**46.2 (22.8)** N = 755	**65.5 (22.1)** N = 681	+42%***	**63.3 (21.4)** N = 581	+37%***	–3%NS
Standard						
*8^th^ Standard*	48.5 (22.5) *n = 182*	54.1 (22.2) *n = 157*	+12%*	57.8 (23.0) *n = 164*	+19%***	+7%NS
*9^th^ Standard*	44.5 (23.0) *n = 284*	67.6 (20.9) *n = 259*	+52%***	64.2 (21.0) *n = 262*	+44%***	–5%NS
*10^th^ Standard*	46.4 (22.7) *n = 289*	70.2 (21.0) *n = 265*	+51%***	67.7 (19.0) *n = 155*	+46%***	–4%NS
Champion status						
*Champion*	43.8 (23.7) *n = 105*	61.2 (23.4) *n = 95*	+40%***	64.2 (23.8) *n = 89*	+47%***	+5%NS
*Non-Champion*	46.6 (22.6) *n = 650*	66.2 (21.9) *n = 586*	+42%***	63.2 (20.9) *n = 492*	+36%***	–5%*
*9^th^ Std. Champion*	44.5 (23.0) *n = 100*	62.0 (22.9) *n = 90*	+39%***	66.1 (23.2) *n = 84*	+49%***	+7%NS
*9^th^ Std. Non-Champion*	44.5 (23.0) *n = 184*	70.5 (19.2) *n = 169*	+58%***	63.3 (19.9) *n = 178*	+42%***	–10%**

*Note*: + denotes positive change, – denotes negative change. *** = *p* < 0.001, ** = *p* < 0.01, * = *p* < 0.05, NS = not significant.

Following the peer-led education sessions, participants demonstrated favorable health attitudes at a rate of 66%, representing a 42% improvement overall, with significant gains across all three standards (*ps* < 0.001 for ninth and tenth standards, *p* = 0.046 for eighth standard) and for both Champions and non-Champions (*ps* < 0.001). Changes in attitudes among older participants were more than four times greater than those demonstrated by eighth standard participants.

Similar to knowledge, scores generally declined between the post-test given immediately after the intervention and at five months post-intervention. However, the overall decline and the declines for the ninth and tenth standard were not statistically significant. Champions and eighth standard participants actually demonstrated positive changes in attitudes between these periods. All scores across all categories at mid-year represented statistically significant increases when compared to baseline scores (*ps* < 0.001).

### Leadership Results

At baseline, most Champions “strongly agreed” with all four statements prior to program participation. At the post-test, even more girls either “agreed” or “strongly agreed” with the statements (Table 4). The only statistically significant improvement was on Question 4 (“I am interested in promoting health knowledge”) (*P* = 0.017 < 0.05).

**Table 4:** Average Leadership Skills Assessment Scores.

	Pre-Test August 2016 mean rating out of 5 (SD)	Post-Test August 2016 mean rating out of 5 (SD)	% change vs. Pre-Test (dir./sig.)

All Champions	N = 105	N = 95	
Q1. I view myself a leader amongst my peers.	4.50 (0.90)	4.64 (0.72)	+3%NS
Q2. I feel confident speaking in front of my classmates.	4.50 (0.90)	4.60 (0.79)	+2%NS
Q3. I believe I can teach and mentor my classmates on important topics.	4.63 (0.68)	4.74 (0.47)	+2%NS
Q4. I am interested in promoting health knowledge.	4.45 (0.84)	4.71 (0.58)	+6%*

*Note*: + denotes positive change, – denotes negative change. *** = *p* < 0.001, ** = *p* < 0.01, * = *p* < 0.05, NS = not significant.

### Final Sample

Participation varied over time due to school absences, participants transferring from or dropping out of participating schools, and academic conflicts. Participants who did not continue participation in the program at mid-year were more likely to be in tenth standard, as the program’s sessions conflicted with preparations in some schools for upcoming standardized exams. Participants who were absent for the administration of the pre-intervention assessment but who were present for the subsequent intervention-related teaching sessions did not complete the immediate post-intervention assessment, but they did complete the mid-year retention Knowledge and Attitude assessments.

## Limitations

Though this study finds positive impacts of the Girls Health Champions intervention, there exist a number of limitations. The primary limitation of this study is that it is a quasi-experimental design. Without a control group, the study cannot attribute changes in knowledge, attitudes, and leadership skills to the intervention alone. For instance, it is possible such evaluation parameters would have naturally shifted over time, or that students may have learned the material covered by Girls Health Champions elsewhere outside of the program. Students may also have become familiar with the assessment following repeated administrations. In order to build on this study, the researchers would consider using a difference-in-difference design, with a control group using the same assessments, to determine whether improvements in knowledge, attitudes, and leadership can be attributed to the Girls Health Champions intervention.

Because students who were lost to follow up on the 5 months post-assessment were largely in tenth standard, and older students tended to have higher assessment scores, differential loss to follow up is possible. Utilizing individual identifiers for participants in the future to more closely track attendance and the completion of assessments would enable researchers to make individual-level assessments of the characteristics and performance of students who drop from the program, thereby potentially strengthening the validity and generalizability of the research.

## Discussion

Our findings from evaluating this peer-to-peer school-based intervention for adolescent Indian girls living in low-income communities in Mumbai include: (1) statistically significant immediate positive impacts on both knowledge levels and attitudes across multiple domains of adolescent health; (2) that such gains were sustained over the academic year; (3) that these benefits accrued to both peer educators and non-peer educator program participants; (4) that the peer educators may have also benefited from positive leadership-related development; and (5) health attitudes are an important and useful evaluation parameter.

Historically, few peer education programs have been evaluated using rigorous techniques. The limited number of evaluations tend to focus on evaluating knowledge levels and use a pre-post design looking at changes in knowledge immediately following the intervention [21,22]. Our study adds evidence to support the efficacy of peer-to-peer training in creating lasting impact on knowledge levels by evaluating responses and assessing retention several months following the intervention. Additionally, few studies have focused on changes in attitudes, both immediately and several months post-intervention. The researchers chose to include attitudes as a research focus given the prevalence in India of deeply entrenched negative attitudes surrounding health topics such as mental health, menstruation, and gender-based violence. Attitudes can serve as precursors to behavior change and are thus important parameters to consider when evaluating peer education programs [23].

Scores on the Knowledge and Attitudes assessments at mid-year, five months after the intervention was conducted, remained statistically significantly higher when compared to baseline scores. For instance, eighth and ninth standard participants scored, on average, over 30% higher on the Knowledge assessment at mid-year than at baseline. On the Attitudes assessment, ninth and tenth standard participants scored over 40% higher at mid-year as compared to baseline. Scores at mid-year did decrease when compared to scores immediately post-intervention, with several months having elapsed since participants were exposed to the intervention’s educational content. Participants might benefit from more frequent sessions to continue building retention over time, and more research could be conducted to better understand the benefits of increasing the frequency of sessions.

Several studies have suggested that peer education programs mainly benefit the peer educators, rather than intended beneficiaries [24,25]. To demonstrate the impact of the intervention on peer educators and non-peer educator participants, the researchers stratified results on the Knowledge and Attitudes assessments by Champion (peer educator) status. The results demonstrate that both Champions and non-Champions benefited from the Girls Health Champions model in terms of statistically significant increases in knowledge levels and statistically significant positive shifts in attitudes, which were sustained over the academic year.

The researchers expanded on existing research by also including leadership skills as a means to measure potential leadership-related impacts on the peer educators. Respondents demonstrated high baseline responses, with all four categories having average baseline scores of 4.5 or greater on a 5-point scale. One category which promisingly demonstrated significant positive change was with regards to the interest level of Champions in promoting health knowledge after they had served as peer health educators. The other three parameters demonstrated positive, non-significant changes, suggesting possible benefits that could be further explored in future studies.

In general, health-related attitudes demonstrated positive shifts, with participants appearing to potentially benefit from repeated exposure to material. For instance, the eighth standard participants and Champions demonstrated higher attitudes scores at mid-year than on the post-test immediately following the initial intervention. This may suggest a dose-response effect whereby participants benefit attitudes-wise following multiple exposures to the intervention. Certain deeply entrenched attitudes proved difficult to modify, suggesting that additional time, sessions, and involvement of parents and community members may be needed to have an impact. For instance, at mid-year, only 34% of girls agreed that “Victims of physical or sexual violence are never at fault or to blame,” compared to 31% at baseline.

Additionally, this study sought to evaluate a more comprehensive approach to peer education that features content addressing many leading causes of adolescent girls’ morbidity and mortality in South Asia. Many youth peer education programs regionally have focused almost exclusively on sexual & reproductive health or early marriage [21,22]. The few peer education programs taking a multi-topic approach were previously unable to demonstrate successful results [26].

The results of this study have important implications in India due to the country’s sizeable adolescent population and recent government-led initiatives to improve adolescent health. In 2014, the Government of India announced Rashtriya Kishor Swasthya Karyakram (RKSK), a national adolescent health program that incorporates out-of-school peer education as part of a multi-pronged strategy to better address the health challenges faced by adolescents in India [16]. The program is currently being implemented across India, and the findings of this study may be useful to those who are developing the peer education component, overseeing the program’s national rollout, or conducting impact evaluations of the program.

This study demonstrates both the potential positive impacts of peer health education programs and the need for more effort and resources to be dedicated to the evaluation, improvement, and implementation of such programs for adolescents worldwide. Adolescents have tremendous untapped potential, and training young people as health educators could help drive long-term gains and shifts in knowledge, attitudes, leadership, and health-related behaviors and practices. The results of this study suggest that a comprehensive, viable school-based peer education model could have positive impacts on adolescent girls, a sizeable population with a tremendous need for health-related education and support both in South Asia and globally.
